# Functional Dissection of the PE Domain Responsible for Translocation of PE_PGRS*33* across the Mycobacterial Cell Wall

**DOI:** 10.1371/journal.pone.0027713

**Published:** 2011-11-16

**Authors:** Alessandro Cascioferro, Maria H. Daleke, Marcello Ventura, Valentina Donà, Giovanni Delogu, Giorgio Palù, Wilbert Bitter, Riccardo Manganelli

**Affiliations:** 1 Department of Histology, Microbiology and Medical Biotechnologies, University of Padua, Padua, Italy; 2 Department of Medical Microbiology and Infection Control, VU University Medical Centre, Amsterdam, The Netherlands; 3 Department of Molecular Microbiology, VU University, Amsterdam, The Netherlands; 4 Institute of Microbiology, Catholic University of the Sacred Hearth, Rome, Italy; Institut de Pharmacologie et de Biologie Structurale, France

## Abstract

PE are peculiar exported mycobacterial proteins over-represented in pathogenic mycobacterial species. They are characterized by an N-terminal domain of about 110 amino acids (PE domain) which has been demonstrated to be responsible for their export and localization. In this paper, we characterize the PE domain of PE_PGRS*33* (PE_Rv1818c_), one of the best characterized PE proteins. We constructed several mutated proteins in which portions of the PE domain were deleted or subjected to defined mutations. These proteins were expressed in different mycobacterial species and their localization was characterized. We confirmed that the PE domain is essential for PE_PGRS*33* surface localization, and demonstrated that a PE domain lacking its first 30 amino acids loses its function. However, single amino acid substitutions in two regions extremely well conserved within the N-terminal domain of all PE proteins had some effect on the stability of PE_PGRS*33*, but not on its localization. Using *Mycobacterium marinum* we could show that the type VII secretion system ESX-5 is essential for PE_PGRS*33* export. Moreover, in *M. marinum*, but not in *Mycobacterium bovis* BCG and in *Mycobacterium tuberculosis*, the PE domain of PE_PGRS*33* is processed and secreted into the culture medium when expressed in the absence of the PGRS domain. Finally, using chimeric proteins in which different portions of the PE_Rv1818c_ domain were fused to the N-terminus of the green fluorescent protein, we could hypothesize that the first 30 amino acids of the PE domain contain a sequence that allows protein translocation.

## Introduction

PE, together with PPE, are peculiar mycobacterial proteins over-represented in pathogenic mycobacterial species. Despite lacking typical secretion signals, both PE and PPE are secreted or located in the mycobacterial envelope [1], [2], [3], [4], [5]. PE proteins can be divided into three distinct subfamilies, of which the most abundant is represented by PE_PGRS. All PE proteins are characterized by an N-terminal highly conserved domain of about 110 amino acids, named PE after the conserved signature motif Pro-Glu (PE) present near the N-terminus. In PE_PGRS proteins, the PE domain is followed by a C-terminal domain with a highly variable Gly-Ala rich sequence [6], [7], which has been suggested to be involved in antigenic variation [8], [9]. In the other PE proteins the PE domain can be followed by an unrelated C-terminal domain, or the PE domain represents the entire protein [7]. In the latter case the PE-encoding gene is usually in tandem with a PPE-encoding gene, and at least in one case the PE and PPE domains encoded by the coupled genes have been shown to interact [10], [11], [12]. Hardly any of the about 100 PE proteins encoded by the *Mycobacterium tuberculosis* genome have been associated with a physiological function, with the exceptions of LipY (Rv3097c), whose C-terminal domain shows lipase activity [13], PE_PGRS*11*, which was recently shown to encode a functional phosphoglycerate mutase [14] and PE_PGRS*33*, which might be involved in induction of macrophage necrosis and apoptosis through interaction with Toll-like receptor 2 [15], [16].

We recently showed that PE_PGRS*33* is surface exposed when expressed in *Mycobacterium smegmatis* and that its PE domain contains the information necessary for the surface exposure. Chimeric proteins based on this PE domain were expressed on the *M. smegmatis* and *Mycobacterium bovis* BCG cell surface [1], [17], [18], and this feature was used to develop a surface delivery system to express heterologous antigen on *M. bovis* BCG envelope and increase its immunogenic potential [18].

As already mentioned, although most PE and PPE proteins lack classical secretion signals, many are exported to the mycobacterial surface, suggesting the involvement of a novel secretory pathway. Interestingly, in *Mycobacterium marinum* their secretion has recently been linked to ESX-5, a member of a novel family of secretion systems typical of mycobacteria (type VII secretion systems) [2], [19], [20].

In this paper, we show that PE_PGRS*33* secretion in *M. marinum* is ESX-5 dependent, and by characterizing the cellular localization of several PE_PGRS*33* mutants and PE-based chimeric proteins in *M. smegmatis*, *M. marinum*, *M. bovis* BCG and *M. tuberculosis* we identify portions of the PE domain that are required for protein translocation.

## Materials and Methods

### Bacterial strains, media and growth condition

*M. smegmatis* mc^2^155 [21], *M. bovis* BCG and *M. tuberculosis* were grown at 37°C. *M. marinum* wild-type strain E11 [22] and its ESX-5 mutant 7C1 [2] were grown at 30°C. All mycobacterial strains were grown in Middlebrook 7H9 broth or on 7H10 solid medium (Difco Becton-Dickinson), supplemented with 0.2% glycerol (Sigma-Aldrich), ADC 10% (Becton-Dickinson), and 0.05% v/v Tween 80 (Sigma-Aldrich). In *M. marinum* secretion experiments, cells grown to mid-logarithmic phase were washed to remove BSA (part of the ADC supplement). Washed cells were inoculated to an OD_600_ of 0.3 in Middlebrook 7H9 supplemented with 0.2% dextrose and 0.05% Tween, and grown for an additional 24 hours. Strains processed for the proteinase K assay or cell fractionation were grown in Sauton's medium (Difco) for 14 hours from a starting OD_600_ of 0.1. For cloning procedures *Escherichia coli* strains HB101 and DH5α were grown in Luria–Bertani medium (LB) [23]. Hygromycin (Roche) was used at a final concentration of 100 µg ml^−1^ (solid media) or 50 µg ml^−1^ (liquid media) for *M. smegmatis*, *M. marinum*, *M. bovis* BCG, and *M. tuberculosis*, and at a final concentration of 200 µg ml^−1^ for *E. coli*. Kanamycin was used at a concentration of 25 µg ml^−1^ for *M. marinum*.

### DNA manipulation

All genes expressed in this work were amplified with Pfu DNA polymerase (Stratagene). Genes encoding HA-tagged proteins were amplified using upper primers designed to contain an NheI site immediately before the start codon, and lower primers designed to contain the HA-coding sequence in frame with the coding sequence of the gene of interest, followed by a stop codon and a BamHI site (Table S1). Mutations in the PE-encoding region were introduced by amplifying the gene with primers containing the mutations (Table S1). The resulting constructs were inserted in the mycobacterial expression vectors pMV10-25 and/or pMV4-36 [24] after NheI and BamHI digestion. For expression in *M. marinum*, fragments containing the P*_hsp60_* or P*_Rv1818c_* promoter sequence, the PE_PGRS*33* encoding gene fragment and the in frame sequence coding for the HA tag were transferred to the pSMT3 vector [20], [25].

To obtain translational fusions between the coding region of the N-terminal part of PE_PGRS*33* and GFP, different portions of the sequence encoding the PE domain of PE _PGRS*33* were amplified using an upper primer containing an XbaI site before the start codon of Rv1818c, and different lower primers containing an in frame XbaI site after the last codon of the PE-encoding region to be included in the construct. The resulting DNA fragments were subsequently cloned in frame with the GFP-encoding gene present in pMV10-25, after digestion with XbaI. Subsequently, DNA fragments containing the promoter and the chimeric genes sequence were extracted from the original plasmids and subcloned in the integrative vector pMV306 [26].

### Electroporation

Electroporation of mycobacteria was performed as previously described [17], [27]. Briefly, cells grown to mid-exponential in phase were extensively washed in 10% glycerol and concentrated approximately 40-fold. 50 or 100 µl concentrated cells were mixed with 1 µg of DNA, and transferred to 0.2 cm gap cuvettes (Eppendorf). Samples were electroporated using an Electroporator 2510 (Eppendorf; capacitance 10 µF; voltage 12.5 kV cm^−1^; resistance 600Ω) (for *M. tuberculosis*), an Electroporator Gene Pulser Transfection Apparatus (Biorad; capacitance 25 µF; voltage 12.5 kV cm^−1^; resistance 200 Ω) (for *M. smegmatis* and *M. bovis* BCG) or a BTX ECM600 Electroporation System (Harvard Apparatus; capacitance 25 µF; voltage 2.5 kV cm^−1^; resistance 720Ω) (for *M. marinum*). After the pulse, the cells were diluted in 900 µl of liquid medium, incubated for 3 h (*M. smegmatis*), 4 h (*M. marinum*) or 24 h (*M. tuberculosis* and *M. bovis* BCG) and then plated on selective solid medium.

### Protein sample preparation

Mycobacterial cells grown to mid-logarithmic phase were separated from culture supernatants by centrifugation. The cells were washed with PBS and thereafter subjected to Proteinase K treatment, Genapol extraction or subcellular fractionation as described below, or sonicated to lyse bacteria. Secreted proteins were precipitated from culture supernatants with 10% TCA (w/v; Sigma-Aldrich). Proteins samples were boiled and separated by SDS-PAGE as described below.

### Proteinase K sensitivity assay

Proteinase K sensitivity assay was performed as previously described [17]. Briefly, selected strains were grown in 20 ml of medium for 14 h starting from an OD_600_ of 0.1. Cells were washed once in TBS buffer (Tris HCl pH 7.5, NaCl 150 mM, KCl 3 mM) and resuspended in 1 ml of the same buffer. Each sample was divided in two identical aliquots, of which one was treated with Proteinase K (Sigma-Aldrich) up to a concentration of 100 µg ml^−1^, whereas the other was left untreated. Both aliquots were incubated for 30 min at 4°C. The reaction was stopped by the addition of 1X complete EDTA-free protease inhibitor (Roche). Subsequently, samples were washed once in TBS and resuspended in TBS plus loading buffer 5X (sucrose 50% w/v, SDS 10% w/v, 0.3 M Tris HCl pH 6.8, bromophenol blue 0.05% w/v, β-mercaptoethanol 5% v/v). Finally, samples were boiled for 10 min to allow bacterial lysis and loaded on a polyacrylamide gel in equal amounts. Treated and untreated samples were processed in parallel using the same procedure to allow their comparison. Each experiment was performed at least twice with different biological samples.

### Genapol extraction of *M. marinum*

Surface-exposed proteins were extracted with the mild detergent Genapol X-080 (Sigma-Aldrich) as previously described [28]. Briefly, intact bacteria were incubated with 0.5% Genapol for 30 min at room temperature with head-over-head rotation. Extracted proteins were separated from the bacteria by centrifugation. Treated cells were lysed by sonication, and all samples were boiled and separated by SDS-PAGE as described below.

### Cell fractionation

Cellular fractionation was performed as previously described [17]. Briefly, 20 ml bacterial cultures were harvested and washed once in 1X PBS at room temperature. The collected culture supernatants were filtered through low protein binding 0.22 µm PVDF filters (Millipore) and concentrated to a final volume of 1 ml on Amicon filters (cut-off 3 kDa) to recover secreted proteins. The pellets were resuspended in 0.5% Genapol X-080 (150 µl/10 mg of wet pellet) and incubated at 30°C for 30 minutes. Extracted proteins were separated from the bacteria by centrifugation. Bacterial pellets were resuspended in PBS 1X/phenyl methane sulphonyl fluoride (Sigma-Aldrich, PMSF) and subjected to sonication. The lysates were centrifuged at 1000 g^−1^ at 4°C to precipitate cellular debris and unlysed cells. Supernatants were transferred to fresh tubes and sedimented at 27.000 g for 30 min at 4°C in order to allow cell wall precipitation. Once again, the supernatant was precipitated at 100.000 g for 2 h to separate cytoplasmic membrane from cytosolic fraction. Cytosolic proteins were subsequently concentrated on Amicon centrifugal filters (cut-off 3 kDa) to a final volume of 1 ml. All sedimented samples were washed once after each step of centrifugation in PBS/PMSF 1 mM and finally resuspended in an appropriate volume of PBS (1 ml) plus Loading buffer 5X. Samples were boiled for 5 min before being separated on polyacrylamide gels and subjected to Western blotting as described below. Protein sorting/localization was calculated by densitometric analysis using the Versadoc Imaging System (Bio-Rad) and Quantity One 4.2.3 software (Bio-Rad).

### SDS–PAGE and Immunoblot

SDS-PAGE was performed according to standard protocols. Briefly, proteins were separated on 10%, 12% or 18% polyacrylamide gels [23], and subsequently transferred to polyvinylidene fluoride membranes (PVDF; Bio-Rad) or nitrocellulose membranes (Amersham Biosciences) by Western blotting. Proteins were visualized by immunoblotting using monoclonal antibodies directed against the HA epitope (Anti-HA.11; Covance, dilution 1∶2000), GFP (Chemicon; dilution 1∶2500), GroEL2 (Rv0440, BEI Resources dilution 1∶200) or ESAT-6 (Hyb 76-8; Statens Serum Institut, Copenhagen, Denmark, dilution 1∶200), and polyclonal antibody directed against a peptide of Rv1698 (Houben et al., in preparation) or against Mpt64 (Delogu et al. in preparation). Secondary goat anti-mouse (Santa Cruz Biotechnology; dilution 1∶2000) or goat anti-rabbit (Santa Cruz Biotechnology; dilution 1∶1000) horseradish peroxidase conjugates were used to detect proteins. The West Dura Signal Kit (Pierce) was used to develop the chemiluminescent signal. Image acquisitions and quantifications were performed using a Versadoc Imaging System (Bio-Rad) and Quantity One 4.2.3 software (Bio-Rad).

## Results

### Construction and expression of different PE_PGRS*33* mutants

**Figure 1:**
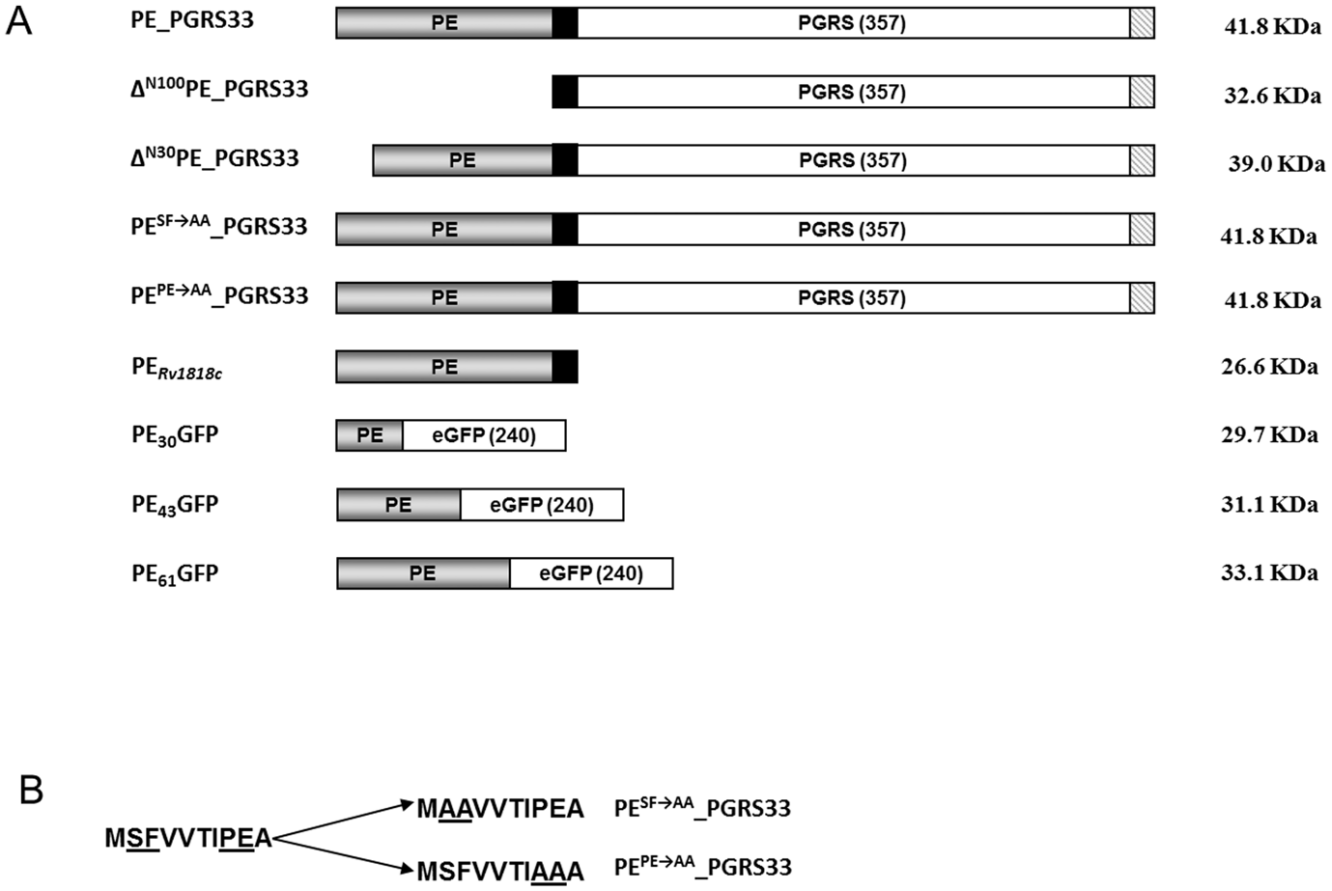
Maps of PE_PGRS*33* and its derivatives used for this study. A) The PE domain is shown in gray, the 41 bp transition domain in black, the PGRS domain and GFP sequence in white, while the 9 amino acid HA epitope is striped; B) sequence of the first 10 amino acids of the wild type PE domain and of the PE domains of the two mutant proteins, in which the conserved SF and PE residues were replaced by alanine residues are shown. Altered amino acids are underlined.

We recently demonstrated that PE_PGRS*33* localizes in the external part of the mycobacterial envelope, and that chimeric proteins in which the PE domain is fused at the N-terminus of an heterologous protein are translocated across the mycobacterial envelope and are surface exposed [17], [18]. Based on these data, which suggest that the PE domain contains a translocation signal, we generated a panel of four mutant PE_PGRS*33* proteins to further characterize the functional role of this domain. As shown in Fig. 1, the first mutant protein (Δ^N100^PE_PGRS*33*) lacked the entire PE domain (100 aa), while the second lacked only the first 30 amino acids (Δ^N30^PE_PGRS*33*). In addition, two mutant proteins that had the same length as wt PE_PGRS*33* were generated, in which either the SF residues at position 2-3 (Δ^SFAA^PE_PGRS*33*) or the PE residues at position 8-9 (Δ^PEAA^PE_PGRS*33*), were replaced by two alanine residues. These SF and PE residues were chosen since they are well conserved across PE domains, suggesting that they may have an important function. All of the mutant proteins were labeled with the HA epitope at their C-terminus to facilitate their detection on Western blots. The genes encoding these proteins were cloned in a replicative plasmid in which they were placed under transcriptional control of the physiological promoter of PE_PGRS*33* (P*_Rv1818c_*). Constructs encoding HA-tagged wt PE_PGRS*33* and an HA-labeled version of its PE domain (PE_Rv1818c_) were available from previous work [17].

### Subcellular fractionation of *M. smegmatis*

**Figure 2:**
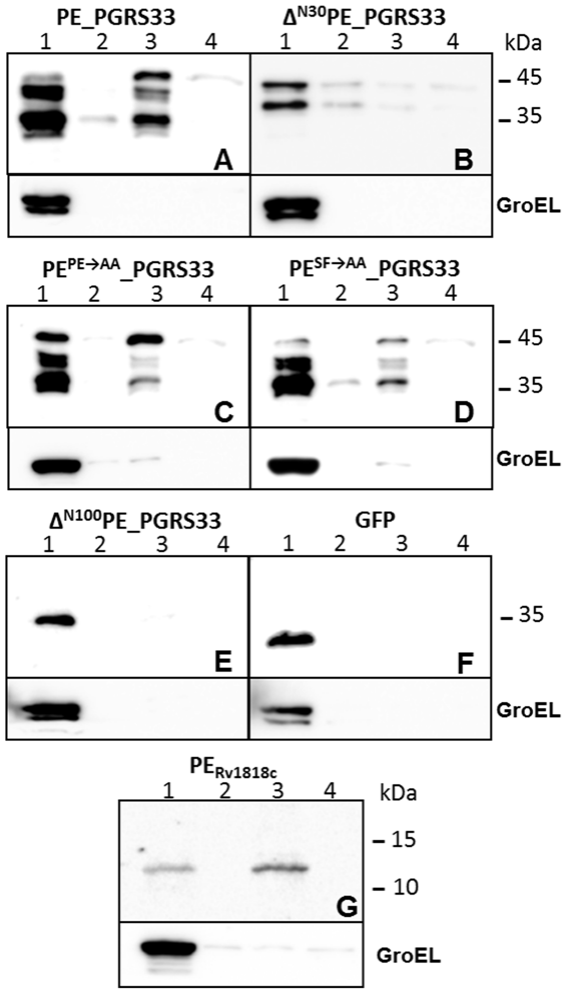
Subcellular fractionation **analysis of** ***M. smegmatis*** **expressing different proteins.** A) PE_PGRS*33*; B) Δ^N30^PE_PGRS*33*; C) PE^PE→AA^_PGRS*33*; D) PE^SF→AA^_PGRS*33*; E) Δ^N100^PE_PGRS*33*; F) GFP; G) PE_Rv1818c_. All constructs were expressed from P*_Rv1818c_* with the exception of that shown in panel B, which was expressed from P*_hsp60_*. Lane 1: cytoplasmic fraction; lane 2: membrane fraction; lane 3: cell wall fraction; lane 4: Genapol supernatant. Proteins were detected by Western blot using monoclonal antibodies against GFP, HA, or GroEL.

The plasmids expressing wt, truncated and mutated forms of PE_PGRS*33* from P*_Rv1818c_* were introduced in *M. smegmatis,* and the subcellular localization of the recombinant proteins was investigated in the resulting strains. Even if *M. smegmatis* genome does not encode PE_PGRS proteins, it encodes a small number of PE proteins and was previously shown to be able to export PE_PGRS*33* [17]. We chose to use a plasmid expressing PE_PGRS*33* from its own promoter, since we previously demonstrated that when this protein was expressed from P*_hsp60_* in *M. smegmatis* it could be found in all fractions due to overexpression [17]. Intact cells were subjected to low temperature extraction with Genapol, a detergent previously shown to extract loosely associated proteins from the mycobacterial surface in these conditions [17], [28], [29], and thereafter a subcellular fractionation procedure was carried out. Cellular fractions were then analysed by Western blot, using GroEL as a control for the cytoplasmic fraction. The results, shown in Fig. 2, confirm that without its PE domain, PE_PGRS*33* remains in the cytoplasm (Fig. 2E). However, the localization of the two proteins carrying the PE and SF mutations did not differ from that of the wt protein (Fig. 2C and D), suggesting that these amino acids, despite their high conservation, do not have a major role in protein localization. Interestingly, the amount of protein extracted with Genapol was extremely low for all proteins, suggesting that previous data showing high level of extraction of this protein in *M. smegmatis* was probably due to overexpression [17]. No PE_PGRS*33* was detected in culture supernatants (not shown). GFP and PE_Rv1818c_ were used as control for cytoplasmic and cell wall proteins, respectively [17], while GroEL was used as a control for cellular integrity. Due to construct instability, it was not possible to obtain a plasmid in which the mutant protein without the first 30 amino acids was placed under P*_Rv1818c_* transcriptional control (not shown). However, we could obtain and express this protein from P*_hsp60_*. As shown in the Fig. 2B, this protein clearly localized in the cytoplasm, although low levels of protein were detectable also in the other fractions (probably due to overexpression).

### ESX-5-dependent PE_PGRS*33* localization in *M. marinum*

**Figure 3:**
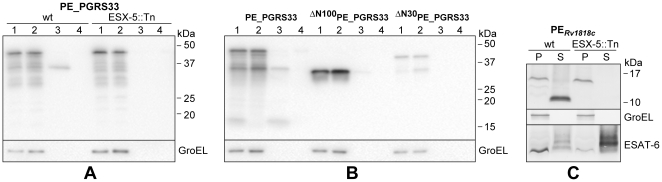
ESX-5-dependent secretion of PE_PGRS*33* in *M. marinum*. Immunoblot analysis of wt and truncated forms of PE_PGRS*33*, expressed from the physiological promoter P*Rv1818c* (A) or P*hsp60* (B-C), in *M. marinum* wt strain E11 and its ESX-5 mutant 7C1 (A and C), or in E11 alone (B). In A-B) fractions containing equal OD units of cell pellets (1), cells treated with Genapol (2), Genapol supernatants (3) and culture supernatants (4) are shown. In C) cell pellet (P) and two-fold more culture supernatant (S) were included. PE_PGRS*33* proteins were detected with the HA antibody, GroEL was included as control for bacterial lysis, and in panel C ESAT-6 was detected as control for protein secretion.

The ESX-5 system has previously been shown to be a major secretion pathway for PE and PPE proteins in *M. marinum* [2], [19], [20]. To investigate whether PE_PGRS*33* is also a substrate of ESX-5, we introduced the plasmid encoding wt PE_PGRS*33* under transcriptional control of its natural promoter in *M. marinum* strain E11 (wild-type) and its ESX-5 mutant 7C1 [2], and analyzed the localization of the expressed proteins. GroEL and ESAT-6 were used as controls for the cytoplasmic and secreted fractions, respectively. As shown in Fig. 3A we could not detect any secretion in the supernatant, but a processed form of PE_PGRS*33* was found in Genapol extracts in the wild-type strain. In the ESX-5 mutant strain this processed form was not present in the Genapol extract, indicating that PE_PGRS*33* is secreted to the bacterial surface by the ESX-5 system of *M. marinum*.

To elaborate on this, we also analyzed a strain expressing only PE_Rv1818c_. In a previous study [17] we showed that in *M. smegmatis* this protein is associated to the cell envelope and that it is not released in the supernatant, even when overexpressed from P*_hsp60_* [17]. However, as shown in Fig. 3C, in *M. marinum* it was substantially released in the supernatant in an ESX-5-dependant manner, confirming ESX-5 dependency and the fact that the PE domain triggers secretion. Interestingly, the secreted protein showed a lower molecular weight suggesting the presence of a maturation process.

Next we tested the effect of the different PE mutations on cell wall localization and secretion. The chimeras carrying the substitution of the PE or the SF residues could not be detected when introduced in *M. marinum* (not shown), suggesting that the PE and SF motifs near the N-terminus of PE_PGRS*33* are important for the stability of the protein in this species. As expected, the proteins lacking either the entire PE domain or its first 30 amino acids were not exported (see Fig. 3B for results with wt *M. marinum*, results for the ESX-5 mutant strain not shown), indicating that this domain is indeed important for secretion.

### Subcellular fractionation of *M. bovis* BCG

**Figure 4:**
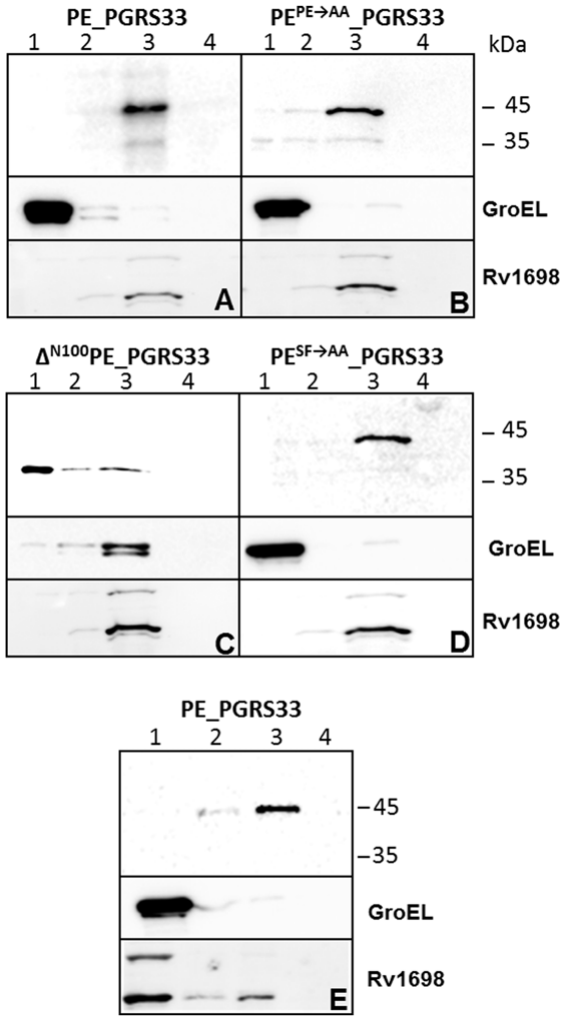
PE_PGRS*33* localization in *M. bovis* BCG and *M. tuberculosis*. Subcellular fractionation analysis was carried out on different cellular fractions of *M. bovis* BCG (A-D) or *M. tuberculosis* (E) expressing PE_PGRS*33* (A and E) or different PE_PGRS*33* mutants (B-D). Lane 1: cytoplasmic fraction; lane 2: membrane fraction; lane 3: cell wall fraction; lane 4: Genapol supernatant. Proteins were detected by Western blot using monoclonal antibodies against, HA, or GroEL, and a rabbit serum against Rv1698

Finally, we decided to study the localization of our chimeric proteins in their natural hosts. For this purpose, the genes encoding the different chimeric proteins under transcriptional control of the PE_PGRS*33* natural promoter (P*_Rv1818c_*) were subcloned in an integrative plasmid, and introduced in *M. bovis* BCG. The resulting strains were subjected to Genapol extraction and subcellular fractionation. Samples were analyzed by Western blot using GroEL and the outer membrane protein Rv1698 as controls for the cytoplasmic and cell wall fractions, respectively [30]. As shown in Fig. 4, the wt protein (Fig. 4A) and its PE and SF mutants localized in the cell wall fraction (Fig. 4B and D), while the Δ^N100^PE_PGRS*33* protein mainly localized in the cytoplasm (Fig. 4C), confirming the results obtained in *M. smegmatis,* with the difference that cell wall localization was more efficient in *M. bovis* BCG. The presence of small amounts of the Δ^N100^PE_PGRS*33* protein in the insoluble fractions, together with that of GroEL in these samples, suggests that the PGRS domain might produce insoluble aggregates that associate with the chaperon GroEL. The construct expressing the protein missing the first 30 amino acids could not be electroporated in *M. bovis* BCG suggesting that this protein is toxic in this species.

The construct expressing the wt protein was also introduced in *M. tuberculosis* and shown to localize entirely in the cell wall fraction (Fig. 4E).

### The PE domain of PE_PGRS*33* is not secreted in *M. bovis* BCG and *M. tuberculosis*

**Figure 5:**
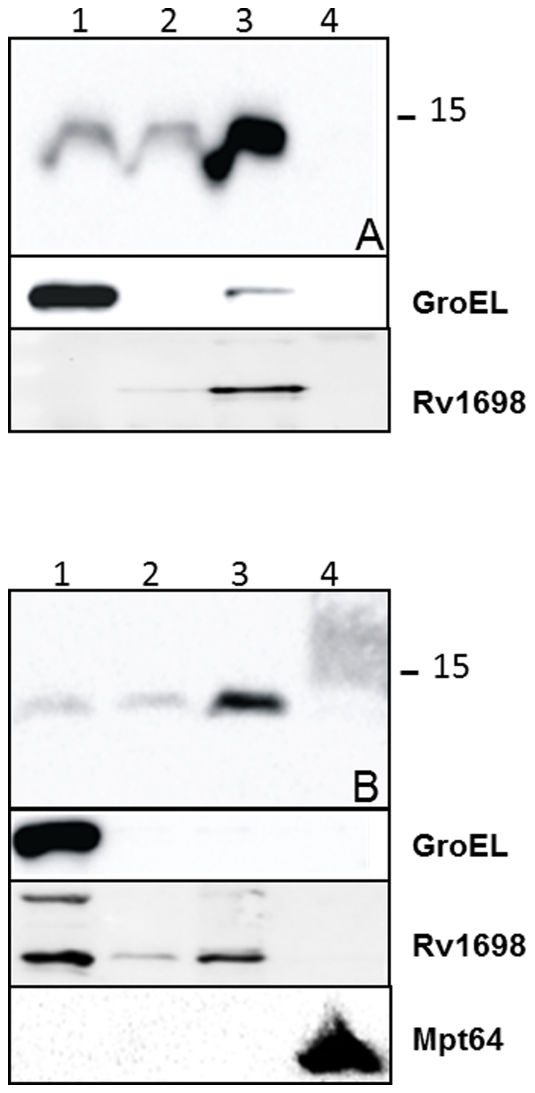
PE_Rv1818c_ localization in *M. bovis* BCG and *M. tuberculosis*. Subcellular fractionation analysis was carried out on different cellular fractions of *M. bovis* BCG (A) or *M. tuberculosis* (B) expressing the PE domain of PE_PGRS*33*: lane 1: cytoplasmic fraction; lane 2: membrane fraction; lane 3: cell wall fraction; lane 4: culture supernatant. Proteins were detected by Western blot using antibodies recognizing HA, Rv1698, GroEL, or Mpt64.

The finding that PE_Rv1818c_ is secreted in *M. marinum* (Fig. 3C) prompted us to study its localization in *M. bovis* BCG and in *M. tuberculosis*, the natural hosts of this protein. However, as clearly shown in Fig. 5, this protein prevalently localized in the cell wall fraction and was not detected in the culture supernatant in these two species, even when overexpressed from P*_hsp60_*. The secreted protein Mpt64 was used as a positive control.

### Identification of a translocation signal in PE_PGRS*33* PE domain

**Figure 6:**
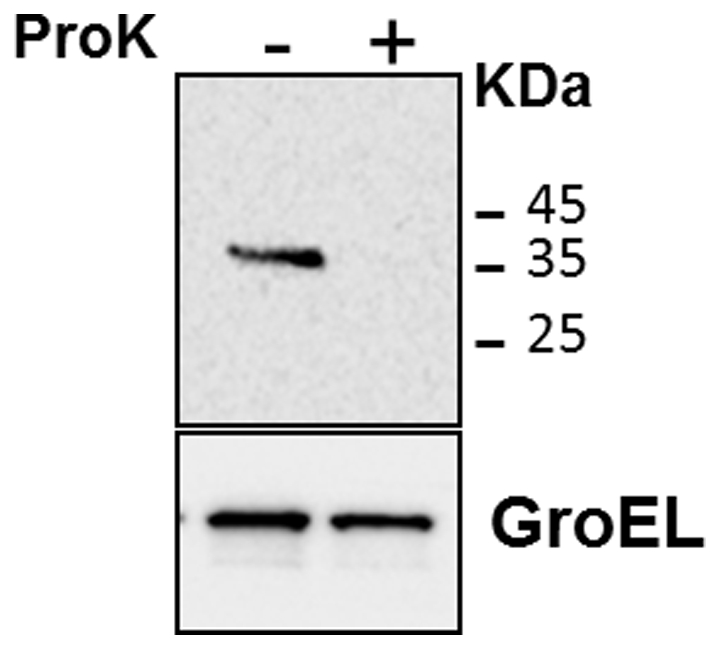
Proteinase K sensitivity assay. The assay was performed on the *M. smegmatis* strain expressing PE_61_-GFP. GroEL was used as a control of cellular integrity. Proteins were detected by Western blot using monoclonal antibodies against GFP or GroEL.

In order to identify the cell wall translocation signal of the PE domain, we constructed three expression plasmids containing translational fusions between the first 30, 43 or 61 aa of the PE_PGRS*33* PE domain and the green fluorescent protein (GFP) under transcriptional control of P*_Rv1818c_* (Fig. 1). Subsequently, the three plasmids were introduced in both *M. smegmatis* and *M. bovis* BCG. The resulting strains were subjected to cell fractionation after Genapol extraction. Even if with different efficiencies, all the constructs were translocated to the cell wall of both species (Fig. S1) suggesting that the first 30 amino acids of the PE domain contain a sequence that allows protein translocation. The *M. smegmatis* strain expressing the PE_61_-GFP chimera was subjected to Proteinase K degradation assay to further prove that this chimera was exposed on the bacterial surface: as shown in Fig. 6, the protein was totally degraded in the presence of the protease, while GroEL (a cytoplasmic protein used as a negative control) was not, demonstrating that the PE_61_-GFP chimera was indeed available on the bacterial surface of *M. smegmatis*.

**Figure 7:**
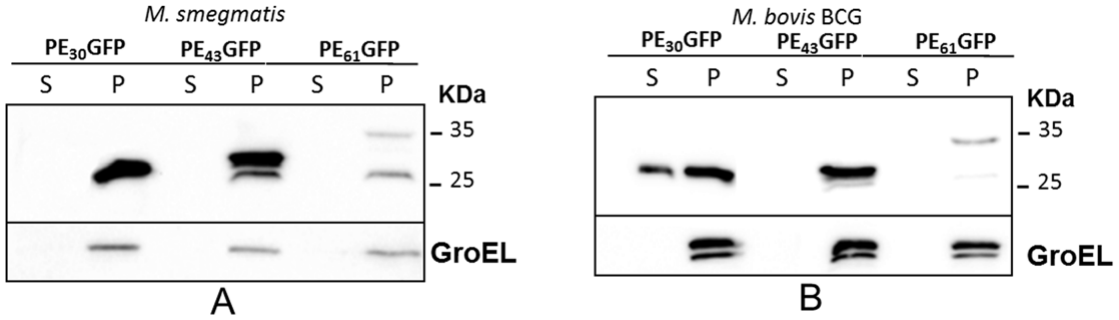
Secretion and surface exposure of PE-based GFP chimeric proteins in *M. smegmatis* and *M. bovis* BCG. Pellet (P) and culture supernatant (S) analysis was carried out on *M. smegmatis* (A) or *M. bovis* BCG (B) strains expressing different fusions of the PE domain of PE_PGRS*33* with GFP. Proteins were detected by Western blot using monoclonal antibodies against GFP or GroEL.

Finally, we analyzed the culture supernatant of these recombinant bacteria. While no recombinant protein was found in the supernatant of the *M. smegmatis* strains (Fig. 7A), surprisingly we found that in *M. bovis* BCG the PE_30_-GFP chimera was partially secreted, (Fig. 7B).

## Discussion

PE are exported mycobacterial proteins characterized by a well conserved N-terminal domain of about 100 amino acids that we recently showed to be required for their export [17], [20]. The lack in the PE domains of clear secretion signals led to the hypothesis that these proteins might be secreted through a new type of mycobacterial-specific secretion system. Indeed, it was recently showed that in *M. marinum* PE protein secretion is abrogated in mutants lacking the type VII secretion system ESX-5 [2], [19], [20]. The aim of this paper is to better characterize the functional portions of the PE domain and their role in translocation through the mycobacterial cell wall. Different PE_PGRS mutant proteins were expressed in different model organisms to study their localization: *i) M. smegmatis*, whose chromosome encodes neither ESX-5 nor PE_PGRS proteins, but was previously shown to be able to export PE_PGRS*33* and chimeric proteins based on its PE domain [17]; *ii) M. marinum*, whose chromosome encodes ESX-5 and many PE_PGRS proteins, but not an apparent PE_PGRS*33* orthologue, and *iii) M. bovis* BCG and *M. tuberculosis*, whose chromosomes encodes both ESX-5 and PE_PGRS*33*. The rationale to use different mycobacterial species was to compare the behaviour of a single PE_PGRS protein in different genetic backgrounds to investigate the conservation of the PE-exporting pathway(s).

As expected, the protein lacking the entire PE domain was not able to translocate and was exclusively found in the bacterial cytoplasm. The same expression profile was found in the mutant protein missing the first 30 amino acids of the PE domain, suggesting that this portion of the protein is essential for the translocation. Surprisingly, mutation of the SF or of the PE conserved residues did not result in any clear phenotype with the exception of a strong instability of the resulting proteins in *M. marinum*.

It is worth noting that PE_PGRS*33* was found in Genapol extracts of *M. marinum*, but not in those of the other tested mycobacterial species suggesting a weaker association of this protein to the cell wall in this species. Of course it is still possible that using different experimental procedures (as increased temperature during extraction) PE_PGRS*33* and/or some of the mutant protein might be extracted with this detergent even in *M. smegmatis, M. tuberculosis* or *M. bovis* BCG.

These results confirm previous proteomics data on Genapol extracts from *M. marinum* as compared to *M. tuberculosis* or *M. smegmatis* [29]. Moreover, the size of PE_PGRS*33* in *M. marinum* Genapol extracts showed a molecular weight lower than that predicted for the entire protein, suggesting a maturation process, as we recently reported for LipY, another PE protein not belonging to the PE_PGRS family [20]. In that case the protein was also processed in *M. tuberculosis*, but only when bacteria were grown inside macrophages, suggesting that still unknown factors only expressed during infection are required for full export and maturation of PE proteins in *M. tuberculosis*. For this reason, PE_PGRS*33* might also be expected to be processed in *M. tuberculosis* during growth inside macrophages. In *M. smegmatis* some of the recombinant proteins showed multiple bands. However, since bands of the same size were present in both cytoplasmic and cell wall fractions, they were likely not due to processing during translocation but rather the result of degradation during cell lysis. The lack of processing observed in *M. smegmatis* might be due to the absence of ESX-5 in this species. Finally, the release of PE_PGRS*33* was totally abrogated in the *M. marinum* ESX-5 mutant, confirming that PE protein translocation in *M. marinum* is dependent on this secretion system.

From previous work [17], we knew that the PE domain of PE_PGRS*33*, when expressed in *M. smegmatis* in the absence of the PGRS domain, localizes in the cell wall without being exposed to the external environment, suggesting that it remains embedded in the mycobacterial outer membrane. Surprisingly, when the same construct was expressed in *M. marinum* it was efficiently secreted in the culture supernatant in an ESX-5-dependent manner, while it remained associated with the cell wall when expressed in *M. tuberculosis* or *M. bovis* BCG. Moreover, in *M. marinum* the secreted form of the PE domain had a lower apparent molecular weight than predicted, indicative of a maturation process, as was previously demonstrated for PE_*PGRS33* wt and for LipY [20].

In the cell fractionation experiments involving *M. tuberculosis* extracts, Rv1698 was not only found in the cell wall fraction (as in those involving *M. bovis* BCG extracts), but also in the cytoplasmic fraction. This imperfect fractionation could be due to the fact that, for biosafety reasons, *M. tuberculosis* was lysed by bead beating instead of sonication.

Since our data suggested that the first portion of the PE domain contains functions required for protein export, we constructed three chimeric proteins in which the first 30, 43 or 61 residues of the PE domain of PE_PGRS*33* were fused to the coding sequence of GFP. All these proteins were able to localize in the cell wall, although in *M. smegmatis* only the construct including the first 61 residues of the PE domain was translocated with a fair efficiency. These result clearly indicated that PE_PGRS*33*-mediated translocation is more efficient in *M. bovis* BCG than in *M. smegmatis*. It should be noted that, while *M. bovis* BCG (as *M. tuberculosis* and *M. marinum*) encodes an ESX-5 secretion system, *M. smegmatis* does not. Moreover, this species encodes only few PE and no PE_PGRS proteins. However, *M. smegmatis* chromosome encodes other type VII secretion systems (as ESX-1 and ESX-3) that might, with low efficiency, complement the absence of ESX-5 and have a role in the secretion of PE_PGRS*33* and its derivatives in this species. Finally, the chimera including only the first 30 amino acids of the PE domain fused to GFP was partially released in *M. bovis* BCG culture supernatant, but not in that of *M. smegmatis*. This interesting finding suggests that the first 30 amino acids of the PE domain contain sufficient information to allow protein translocation. The only structural information available for a PE protein derives from PE25, a protein including only the PE domain, whose structural gene is followed by the gene encoding a protein of the PPE family (PPE41). These two proteins were shown to interact to form a heterodimer, for which the crystal structure was solved [10]. The PE structure included two antiparallel α-helices (the first between residues 8 and 37, the second between residues 45 and 84) connected by a loop (residues 38-44) [10]. If the different PE domains have a similar folding, the first chimera (PE_30_-GFP) would include most of the first α-helix, while the second (PE_43_-GFP) would include both the first α-helix and the loop, and the last chimera (PE_61_-GFP) would include the first α-helix, the loop and part of the second α-helix (Fig. S2). Our data suggest an involvement of the first α-helix in directing the protein to the secretion system. The fact that the other two chimeric proteins were not secreted and were detected in the cell wall fraction, suggests that the loop between the two α-helices might be involved in the association of the PE domain with the cell wall, even though no similarity with cell wall anchoring domains of other bacteria was identified. However, it is also possible that the PE_43_-GFP and the PE_61_-GFP chimeric proteins form insoluble aggregates that co-localize with the cell wall, or that they are misfolded and bind the membrane via exposed hydrophobic patches.

The identification of an N-terminal portion of a PE domain responsible for translocation and association to the bacterial surface opens new avenues to study the interaction of type VII substrates and the secretion machinery and could be used to identify novel substrates. The fact that at least one of the ESX-1 substrates (Cfp10) was shown to have a C-terminal signal peptide [31] opens the interesting possibility that multiple secretion signals might be recognized by type VII secretion system and be present in their targets.

## Supporting Information

Figure S1 **Localization of PE-based GFP chimeric proteins in** ***M. smegmatis*** **and** ***M. bovis*** **BCG.** Subcellular fractionation analysis was carried out on different cellular fractions of *M. smegmatis* (A-D) or *M. bovis* BCG (E-H) expressing GFP or different fusions of the PE domain of PE_PGRS*33* with GFP: lane 1: cytoplasmic fraction; lane 2: membrane fraction; lane 3: cell wall fraction; lane 4: Genapol supernatant. Proteins were detected by Western blot using monoclonal antibodies against GFP.
(TIF)

Figure S2 **Putative structure of the PE domain of PE_PGRS** ***33*** **and of the chimeric proteins in which small fragments of the PE domain were fused to GFP.** The figure was drawn assuming that the PE domain of PE_PGRS*33* has a structure similar to the PE domain of PE25 [10].
(TIF)

Table S1 Primers used in this study.
(DOC)
