# Supplementary figures and images for: Functional Dissection of the PE Domain Responsible for Translocation of PE_PGRS33 across the Mycobacterial Cell Wall
Source: PLoS One. 2011 Nov 16;6(11):e27713. doi: 10.1371/journal.pone.0027713 (PMC3218021; doi:10.1371/journal.pone.0027713)

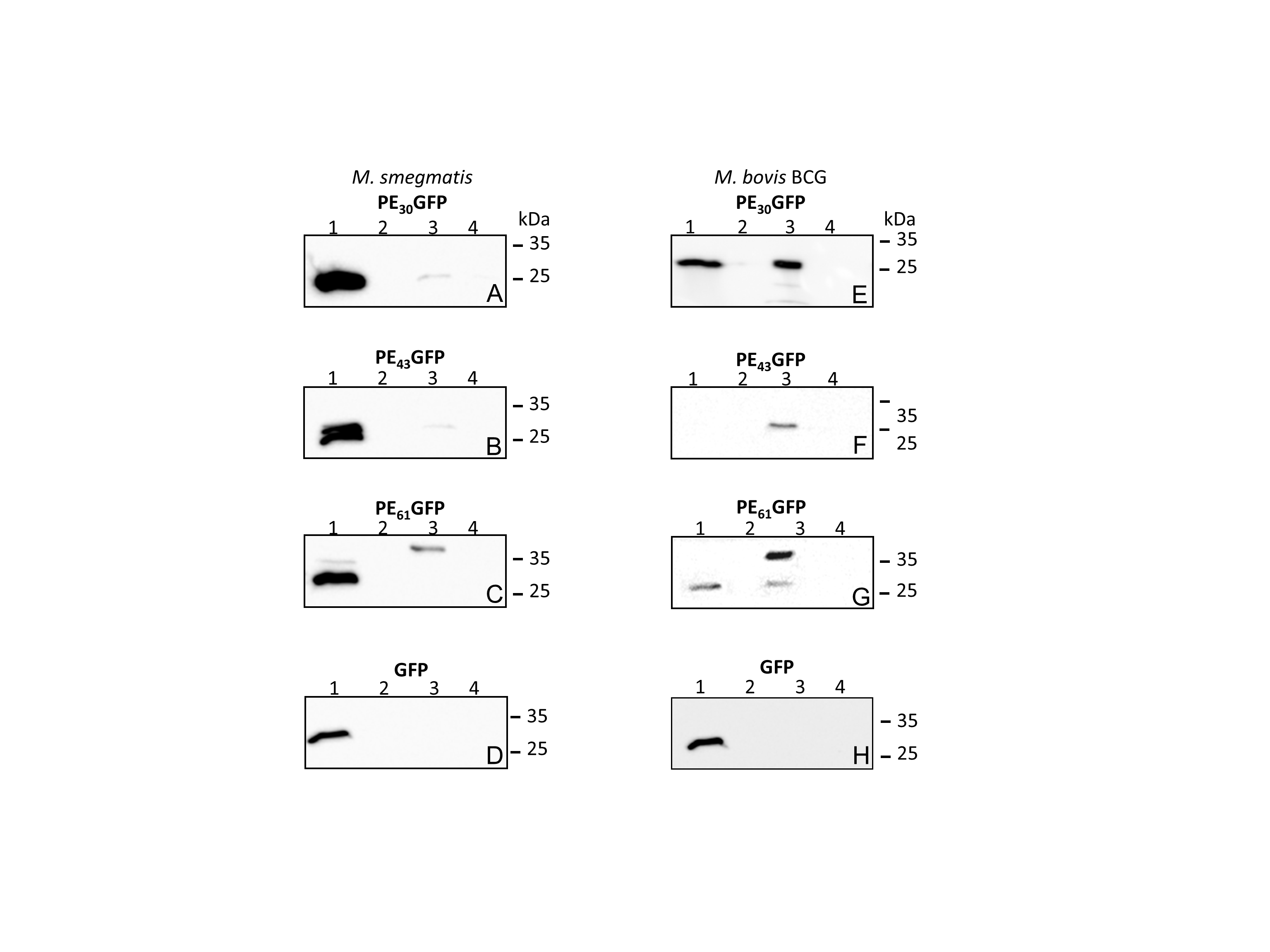

Supplement: Figure S1 — Localization of PE-based GFP chimeric proteins in M. smegmatis and M. bovis BCG. Subcellular fractionation analysis was carried out on different cellular fractions of M. smegmatis (A-D) or M. bovis BCG (E-H) expressing GFP or different fusions of the PE domain of PE_PGRS33 with GFP: lane 1: cytoplasmic fraction; lane 2: membrane fraction; lane 3: cell wall fraction; lane 4: Genapol supernatant. Proteins were detected by Western blot using monoclonal antibodies against GFP. (TIF) [file pone.0027713.s001.tif]

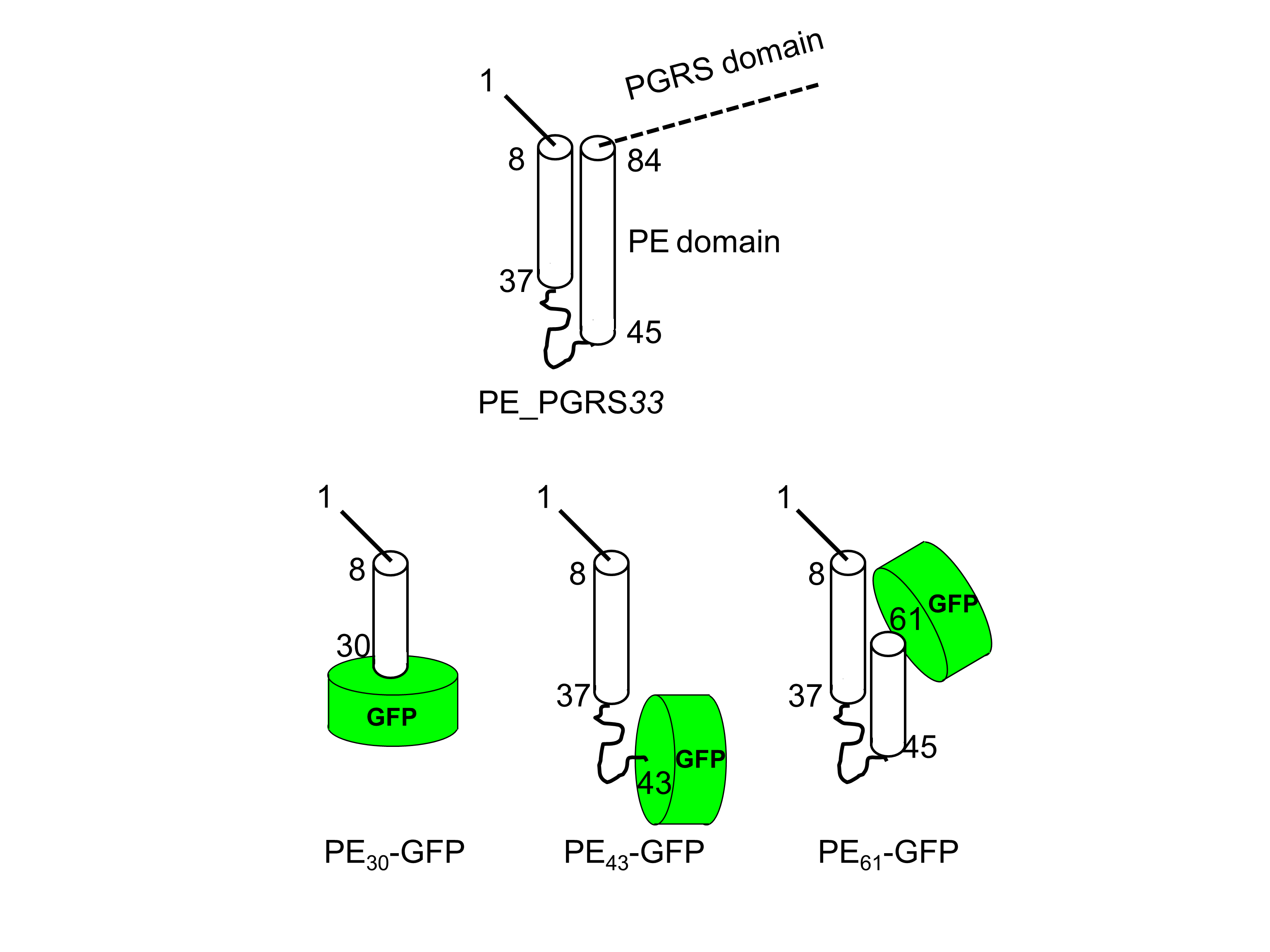

Supplement: Figure S2 — Putative structure of the PE domain of PE_PGRS 33 and of the chimeric proteins in which small fragments of the PE domain were fused to GFP. The figure was drawn assuming that the PE domain of PE_PGRS33 has a structure similar to the PE domain of PE25 [10]. (TIF) [file pone.0027713.s002.tif]
