# Supplementary material for: Functional Dissection of the PE Domain Responsible for Translocation of PE_PGRS33 across the Mycobacterial Cell Wall
Source: PLoS One. 2011 Nov 16;6(11):e27713. doi: 10.1371/journal.pone.0027713 (PMC3218021; doi:10.1371/journal.pone.0027713)
Supplement: Table S1 — Primers used in this study. (DOC) [file pone.0027713.s003.doc]

| **Primer** | **Sequence^a^** | **Product^b^** |
| --- | --- | --- |
| RP677 | AAAGCTAGCATGGTGCTCGACGTGATCAACGC | *Δ^N100^* PE_PGRS*33*-HA (U) |
| RP679 | ATGGATCCTTA*AGCATAATCAGGAACATCATATGGATA*GGGTAACCCGTT | *Δ^N10 0^* PE_PGRS*33*-HA (L)  *Δ^N30^* PE_PGRS*33*-HA (L)  *Δ* ^PE→AA^ PE_PGRS*33*-HA (L) |
| RP711 | AAAGCTAGCATGGCCGCCGCGGTCCCGAC | *Δ^N30^* PE_PGRS*33*-HA (U) |
| RP679 |  | *Δ^N30^* PE_PGRS*33*-HA (L) |
| RP680 | TTTCTAGAATG**G**CA**GC**TGTGGTCACGATCCCGGAGGC | *Δ* ^SF→AA^ PE_PGRS*33*-HA (U) |
| RP681 | TTTCTAGAATGTCATTTGTGGTCACGATT**G**CGG**C**GGCACTAGCGGCGGTG | *Δ* ^PE→AA^ PE_PGRS*33*-HA (U) |
| RP712 | AGTCTAGAATGTCATTTGTGGTCACGAT | PE_30_ (U)  PE_43_ (U)  PE_61_ (U) |
| RP1072 | AGTCTAGAAGCGTTGGCGGTGCCGAT | PE_30_ (L) |
| RP1073 | AGTCTAGAGGCGGCGGCCAACACC | PE_43_ (L) |
| RP1074 | AGTCTAGAGGCCTGGGCGTGTCCG | PE_61_ (L) |

**Table S1. Primers used in this study.**

^a^ Restriction sites are underlined, nucleotides encoding the HA epitope are in italic, mutated codons are in bold.

^b^ Protein encoded by the gene amplified by the corresponding primers. U: upper primer; L: lower primer
